# Evaluation of the dispersion of metakaolin–graphene oxide hybrid in water and cement pore solution: can metakaolin really improve the dispersion of graphene oxide in the calcium-rich environment of hydrating cement matrix?†

**DOI:** 10.1039/d1ra01504d

**Published:** 2021-05-24

**Authors:** Kasra Amini, Siavash Soleimani Amiri, Ali Ghasemi, Sajjad Mirvalad, Asghar Habibnejad Korayem

**Affiliations:** School of Civil Engineering, Iran University of Science and Technology Tehran Iran mirvalad@iust.ac.ir ahkorayem@iust.ac.ir

## Abstract

Graphene oxide (GO) is a promising candidate for reinforcing cement composites due to its prominent mechanical properties and good dispersibility in water. However, the severe agglomeration of GO nanosheets in the Ca^2+^ ion loaded environment of a freshly mixed cement composite is the main obstacle against the mentioned goal. Recent studies, based on the SEM images, have shown that the incorporation of pozzolans can ameliorate the GO agglomeration in cement matrix. Considering the fact that, for identifying the GO dispersion in cement matrix, SEM characterization is not preferred due to the hydrated cement matrix complexity and presence of small dosages of GO, this research has investigated the potential of Metakaolin (MK) as a highly reactive pozzolan against GO agglomeration in the non-hydrated environment of simulated cement pore solution (SCPS) for different MK/GO weight ratios. Additionally, the interaction between MK and GO in water is evaluated through different characterization methods. Visual investigation and UV-vis spectroscopy revealed that there should be a probable interaction between MK particles and GO nanosheets in water which was interpreted by Lewis acid–base interaction and further examined by FTIR spectroscopy. Moreover, the zeta potential measurements indicated that the increase in MK/GO weight ratio could lead to higher adsorption of GO on the surface of MK particles which was confirmed by the particle size analysis. Almost all of the conducted experiments on the MK–GO hybrid in simulated cement pore solution showed that different dosages of MK particles were incapable of preventing GO agglomeration; thus, despite the proposed mechanisms in previous studies, MK cannot effectively restrict the unfavorable effects of Ca^2+^ ions on GO dispersion in SCPS and analogously in the hydrating cement matrix.

## Abstract 2

The aggregation mechanism of an MK–GO hybrid in the pH range of 10–11 due to the surface complexation between Al(OH)_4_^−^ formed by MK dissolution and the graphene layer of GO through Lewis acid–base interaction.

## Graphical abstract

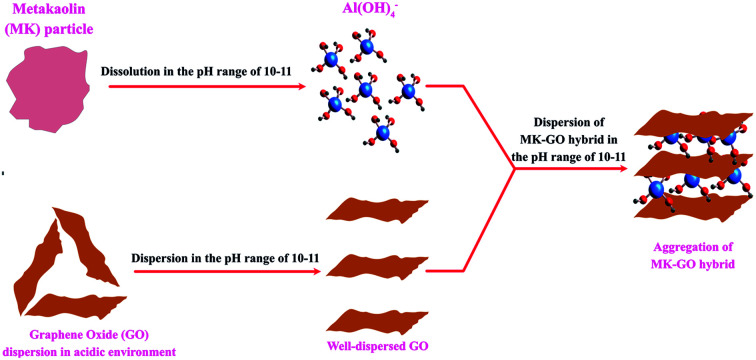

## 1. Introduction

In recent years, with the increasing developments in nanotechnology, many studies have been devoted to the use of graphene and its derivatives such as graphene oxide (GO) in cement composites. On the positive side, GO has the ability to promote the mechanical and microstructural properties of cement composites due to its unique mechanical properties, high surface area, and good dispersibility in water.^1–10^ For instance, with 0.05% of GO incorporation in mortar samples, the 28 days compressive and tensile strength was increased by 41% and 25%, respectively, compared to the plain sample.^11^ Also, the reinforced cement paste sample with GO dosage of 0.05% showed Young's modulus approximately 18% higher than the reference one.^12^ On the negative side, the main challenge that may limit the use of GO arises from its dispersion status in cementitious host matrices as the GO nanosheets tend to agglomerate owing to intense van der Waals forces among them coupled with their high surface area.^13,14^ By the formation of these agglomerates and subsequently increasing their size, the formation of weak zones and stress concentration in cement composites would be probable which degrades the effectiveness of using GO nanosheets.^15–17^ As reported by Lu *et al.*,^17^ the well-dispersed GO was able to improve the compressive strength of cement paste by 11.9%, while the aggregated GO had a marginal effect on enhancing the compressive strength.

More specifically, from chemical viewpoint, in the case of GO in cementitious environments, the presence of large amounts of alkali metal cations such as Na^+^, K^+^, and Ca^2+^ leads to GO agglomeration. As Ca^2+^ has higher interaction energy with GO functional groups in comparison with Na^+^ and K^+^, it has a key role in the agglomeration of GO nanosheets.^18^ Another possible mode of the effect of Ca^2+^ on agglomeration is the cross-linking of GO nanosheets *via* interaction with carboxyl groups at the edges of the basal plane.^19^ Besides the influence of metal cations, the pH of cement pore solution is an important factor that influences the GO dispersion. In a high alkaline medium, the hydrophilic functional groups of GO will be detached from its surface and GO agglomerates.^18^

However, a controversial solution to facilitate the GO dispersion in high alkaline environments is the incorporation of GO along with pozzolanic materials such as silica fume or fly ash, which has been investigated mainly into the cement composites. Li *et al.*^20^ reported that an optimized amount of silica fume can cover the surface of GO sheets and prohibit the interaction between GO functional groups and the existing ions in the pore solution. They also stated that silica fume consumes Ca(OH)_2_ that is produced by cement hydration through the pozzolanic reaction and reduces the concentration of Ca^2+^, thus preventing GO agglomeration. The analogous result has been reported by Indukuri *et al.*^21^ By comparison the SEM images of GO modified cement pastes with/out silica fume after 28 days of hydration, they declared that silica fume can mechanically separate GO nanosheets from divalent calcium ions. With regard to fly ash, Wang *et al.*^22^ revealed that fly ash particles can improve GO dispersion in cement paste samples by breaking up the agglomerations of GO–cement particles. This conclusion was based on the characterizing of the rheological properties of GO–cement and fly ash–GO–cement pastes up to 120 min since their fabrication. In contrast to the abovementioned studies, some chemical experiments were carried out by Lu *et al.*^23^ in order to comparatively investigate the dispersion of GO in aqueous and alkaline cementitious solutions by incorporating silica fume. They reported that despite the positive effect of silica fume on GO dispersion in water, the incorporation of silica fume worsens the dispersion of GO in cement pore solution. Accordingly, they announced that their findings were opposite to the results of the previous studies, revealing the necessity of performing further researches.

The dispersion morphology of GO in natural aqueous environments and the subsequent environmental impact has been attracted more attention currently due to the wider applications of GO in various fields and consequently the higher level of GO production, which indicates a greater risk of environmental exposure to GO.^24^ However, the probable interactions between GO and different minerals present in aquatic environments play a key role in the dispersibility of GO.^25^ Among these minerals, clay-based minerals such as kaolin and montmorillonite have great importance for investigation mainly owing to their massive use in industrial applications,^26^ which signifies the potential of their release into the aquatic environments. Regarding kaolin minerals, it was found that the kaolin nanoclay can effectively alleviate the GO toxicity in aqueous mediums by strongly coagulating with GO, thus producing relatively large conglomerates. Indeed, the increase in the hydrodynamic diameter of the resulting coagulated kaolin–GO hybrid compared to pure kaolin or GO elucidated the aggregation of GO in the presence of kaolin particles.^27^ The adsorption of GO nanosheets by kaolin particles and their consequent coagulation in aqueous environments was confirmed later in a study performed by Rozhina *et al.*^28^ Their results of AFM topography images coupled with the adhesion force maps of kaolin, GO, and kaolin–GO samples demonstrated the deposition of GO nanosheets onto the surface of kaolin particles.^28^ Moreover, the interaction between kaolin and GO in aqueous environments was further investigated by Hoor *et al.*^29^ By comparison the FTIR spectra of kaolin, GO, and kaolin–GO hybrid, they declared that the adsorption of GO on the kaolinite particles was responsible for diminishing the original functional groups of kaolin such as O–H and Si–O and substituting them with the oxygen-containing functional groups of GO. Also, as compared to pure kaolinite suspensions, a shift in the magnitude of zeta potential of kaolinite–GO suspensions at all pH regions was found, which was mainly attributed to the adsorption mechanism of GO on kaolin.^29^ However, the aforementioned results were in contrast to those of Huang *et al.*^30^ and Zhao *et al.*,^31^ who reported that GO had no or minimal adsorption toward kaolinite particles due to the electrostatic repulsion between them, which was based on the negative zeta potential values of both GO and kaolinite. On the other hand, in the case of montmorillonite minerals, Ge *et al.*^32^ found that the restacking of reduced graphene oxide (rGO) nanoparticles could be considerably prevented by the application of montmorillonite nanosheets.

With regard to the application of clay-based minerals in cement composites, their capability to disperse carbon nanostructures like carbon nanotube (CNT) or GO is increasingly gaining popularity among researchers. For instance, Morsy *et al.*^33^ reported the potential of nano metakaolin (NMK) particles for CNT dispersion in mortar composites by disrupting the fiber–fiber interactions (in terms of van der Waals forces) between the CNT clumps. Indeed, due to the extremely smaller size of NMK particles compared with that of anhydrous cement grains, NMK particles were able to be situated between the CNT clumps during the procedure of dry mixing, thus causing the separation of CNT fibers. However, unlike the outcomes of the aforementioned study, Neto *et al.*^34^ showed that the incorporation of 0.1% CNT into blended metakaolin mortars led to an increase in porosity and sorptivity, along with the reduction of tensile and compressive strength. Moreover, they indicated that compared to the unblended mortars, the effects of CNT agglomeration were more intense in blended ones. The lower efficiency of CNT addition to improve the mechanical properties of blended metakaolin mortars was attributed to the hindering effects of metakaolin on CNT dispersion and/or *vice versa*. In the case of GO, Roy *et al.*^35^ employed metakaolin/silica fume hybrid to enhance GO dispersion and consequently promote the macroscopic properties of GO reinforced cement mortars. Although they reported better mechanical and transport properties due to the incorporation of metakaolin/silica fume hybrid, they did not conduct any specific dispersion experiment to illuminate the effects of metakaolin/silica fume hybrid on the quality of GO dispersion. Furthermore, their findings were mainly based on the SEM images and macroscopic properties of the hydrated cement composites. Since the effect of silica fume on GO dispersion has been profoundly investigated, it seems that there is a gap of knowledge in evaluating the influence of metakaolin on the dispersion of GO nanosheets in non-hydrated mediums using a combination of the related experiments. The main aim of these experiments should be to characterize the GO dispersion in the non-hydrated environment of the freshly mixed cement composites rather than SEM imaging or macroscopic investigation of the GO modified hydrated samples.

In fact, as no significant change in dispersion morphology of GO can be expected after setting and consequent hardening of cement-based composites, it is so important to monitor the quality of dispersion at a very early age of hydration. This way, a more realistic judgment about the capability of various kinds of pozzolans against GO agglomeration can be made. Therefore, in this study, through the incorporation of different MK/GO weight ratios that are frequently used in practice, the effect of MK on the dispersion of GO in water and simulated cement pore solution was analyzed by conducting pH measurements, UV-vis spectroscopy, scanning electron microscopy (SEM) imaging, laser diffraction (LD), zeta potential measurement, and dynamic light scattering (DLS). Furthermore, in order to examine the interaction mechanism between GO and MK in water, Fourier-transform infrared spectroscopy (FTIR) was employed. The results of this study provide new insights into the interaction between pozzolans and GO in water and cementitious environments such as the hydrating cement matrix.

## 2. Materials and methods

### 2.1. Raw materials

The used metakaolin in this study was supplied by a local producer. The chemical composition of the as-received metakaolin was characterized by X-ray fluorescence (XRF) test. In addition, the specific gravity and fineness of metakaolin were determined according to the procedure prescribed by ASTM C 311/C 311M-13 and ASTM C 204-16, respectively. The chemical composition and physical properties of metakaolin are listed in Table 1. The particle size analysis was performed on metakaolin through the laser diffraction method; the particle size distribution graph is shown in Fig. S1(a).† Also, the SEM image of metakaolin solid particles is presented in Fig. S1(b).† Moreover, the X-ray diffraction (XRD) analysis was conducted on a sample of metakaolin to characterize its principal mineral phases. The XRD pattern of metakaolin is depicted in Fig. S1(c).† Besides, GO suspension with the concentration of 1 mg ml^−1^ was purchased from NAMAGO Company (Iran). The elemental analysis of the as-received GO suspension is shown in Table 2. Additionally, the SEM image of GO nanosheets is exhibited in Fig. S2(a).† As can be seen clearly, the wrinkled morphology of GO nanosheets with a huge surface area was the prominent characteristic that was observed in the SEM image. For further identifying the as-received GO suspension, the XRD analysis was performed in the wide scanning 2*θ* range of 5° to 70° by a diffractometer (Bruker, advance-D8) using Cu Kα radiation (*λ* = 0.154 nm). Fig. S2(b)† shows the XRD pattern of GO. It can be seen that GO has a unique and major peak at 2*θ* ∼ 10.96°. On the basis of this result, the interlayer spacing between graphene layers in GO was calculated using the Bragg's equation that was equal to *d* ∼ 0.81 nm. Besides, the chemical bonds and functional groups of GO were investigated *via* Fourier-transform infrared (FTIR) spectroscopy which was performed on a dried sample of GO suspension. Fig. S2(c)† illustrates the FTIR spectrum of GO. Seven featured peaks are demonstrated in the FTIR spectrum of GO. The broad peak at 3425 cm^−1^ was attributed to the hydroxyl group (–O–H). The carbonyl group (C=O) and aromatic (sp^2^ hybridized carbon) (C=C) peaks were observed at 1740 cm^−1^ and 1625 cm^−1^, respectively. Also, the peaks at 1379 cm^−1^, 1260 cm^−1^, and 1074 cm^−1^ indicated the existence of carboxy (alkoxy) (C–O), epoxide (C–O–C), and hydroxyl (C–OH) groups in the GO structure. In addition, it should be noted that the peaks at 2926 cm^−1^ and 2859 cm^−1^ may be due to the presence of C–H bond in the chemical structure of GO. Because of the existence of the aforementioned oxygen-containing functional groups in the FTIR spectrum of GO, it can be concluded that GO has a hydrophilic nature and can be well dispersed in water.

**Table 1:** Chemical composition and physical properties of metakaolin

Material	Chemical composition (%)									Physical properties	
	SiO_2_	Al_2_O_3_	CaO	Fe_2_O_3_	SO_3_	MgO	K_2_O	Na_2_O	LOI	Specific gravity (g cm^−3^)	Blaine (cm^2^ g^−1^)
Metakaolin	74.23	15.33	5.05	1.01	0.27	0.18	0.62	—	2.92	2.40	3818

**Table 2:** Elemental analysis of as-received GO suspension

Element	Carbon (C)	Oxygen (O)	Nitrogen (N)	Sulfur (S)
Percentage (%)	58–63	33–38	0–2	1–2

### 2.2. Fabrication of simulated cement pore solution (SCPS) as the cementitious environment

To investigate the dispersion of MK–GO suspensions in an environment representing the medium of the freshly mixed cement composites, SCPS was fabricated according to the instruction recommended by Ghods *et al.*,^36^ as shown in Table S1.† For preparing the SCPS, the powders of Ca(OH)_2_ and CaSO_4_·2H_2_O as well as the NaOH and KOH pellets were poured into a container of deionized water (pH = 6.7) and then were stirred with a magnetic mixer for 5 minutes at a rotational speed of 1000 rpm. Next, the solution was filtered twice to make it transparent and free of any solid particles. The ICP-OES (Varian, 730-ES) was employed to characterize the elemental concentration of the prepared SCPS. The results are listed in Table S2.† Also, it should be pointed out that the pH of SCPS ranged from 13.3 to 13.5.

### 2.3. Mix designs of dispersion experiments

According to the frequently utilized proportions of GO and MK by weight of cement, six MK/GO weight ratios including 100, 300, 600, 1000, 1500, and 2000 were chosen for various dispersion experiments. Besides the evaluation of the interaction between MK and GO in water, the main purpose of these characterizations was to examine the possibility of the existence of an optimum MK/GO weight ratio to ameliorate the GO dispersion in high alkaline cementitious environments. It should be pointed out that in order to make the results of the MK–GO suspensions comparable, two extra mixes individually containing MK and GO as reference mixes were also prepared. Additionally, the amount of suspensions' volume was determined based on the requirements of the various dispersion experiments. Before conducting dispersion experiments, each suspension was ultrasonicated for 10 min at the power of 50 W. The mix designs of MK, GO, and MK–GO for dispersion experiments in water and SCPS are shown in Table 3.

**Table 3:** Mix designs of MK, GO, and MK–GO samples in water and SCPS

Sample number	Compound (mg)		Sample volume (mL)	Host matrix
	MK	GO		
1	—	2	50	Water/SCPS
2	1200	—	50	Water/SCPS
3	200	2	50	Water/SCPS
4	600	2	50	Water/SCPS
5	1200	2	50	Water/SCPS
6	2000	2	50	Water/SCPS
7	3000	2	50	Water/SCPS
8	4000	2	50	Water/SCPS

### 2.4. Characterizations

#### 2.4.1. pH measurements

A glass electrode (Ionode, IH-40A) pH meter (EZDO, PL-700 PV) was used to determine the pH values of MK–GO suspensions in water as well as their pH variation in SCPS, during the initial hours since their fabrication. In water, the corresponding weight of MK was added to the diluted GO suspension (50 ml) and then mixed for 10 min prior to the pH measurement. However, in SCPS, initially, a specific volume of pre-mixed MK–GO suspension (25 ml) was added to SCPS (25 ml), and then the resulted suspension was mixed for 10 min. Moreover, before the pH measurements, the pH meter was calibrated using the standard buffer solutions with a pH of 4.01 and 7.00. Finally, it is noteworthy mentioning that for each MK/GO weight ratio, the average value of three pH measurements lasting 30 min was reported as the pH value of the corresponding suspension.

#### 2.4.2. UV-vis spectroscopy

The effect of MK on GO dispersion was determined through UV-vis absorption over the wavelength ranged from 200 to 700 nm with an accuracy of 0.3 nm by means of a high performance spectrophotometer (PG Instruments, T80+). It is worth mentioning that prior to the experiment, all of the suspensions were centrifuged for 6 min at a rotational speed of 12 000 rpm to separate their solid particles. All of the samples had the same GO concentration equals to 0.04 mg ml^−1^ in both mediums of water and SCPS. Moreover, all of the UV-vis absorption experiments were conducted on a quartz cuvette containing 3 ml of the samples.

#### 2.4.3. Zeta potential

The zeta potential measurement was performed using a zeta potential analyzer (Horiba Jobin Yvon, SZ-100z) to examine the interaction between MK and GO in water and SCPS. The sodium carbonate–sodium bicarbonate buffer solution was used to adjust the pH of MK–GO hybrid suspensions in water due to their moderate alkalinity. Moreover, because of the high alkalinity of SCPS, the pH of the suspensions in this medium was adjusted by KOH solution. The zeta potential value of each suspension was reported based on the mean value of three replications.

#### 2.4.4. Particle size distribution analysis

Two different techniques were used to investigate the particle size distribution of the dispersion mixtures in water and SCPS. Dynamic light scattering (DLS) (Horiba Jobin Yvon, SZ-100z) was employed to determine the particle size of GO dispersion in water. Since the lateral size of GO agglomerates in SCPS was reported approximately in the range of a few micrometers up to hundreds of micrometers,^37^ the laser diffraction (LD) technique (Sympatec Helos, H2396) was adopted for analyzing the lateral size of GO aggregates in SCPS. In addition, the particle size of all the mixtures containing metakaolin was characterized by LD technique.

#### 2.4.5. SEM characterization

A field emission scanning electron microscope (JEOL, 7001F FEG) was used to take SEM micrographs of MK–GO suspensions in both water and SCPS. Prior to the SEM imaging, the sample was deposited on a silicon wafer and after drying, a thin layer of platinum was coated to make the sample conductive. Also, the elemental analysis of each sample was carried out using the energy-dispersive X-ray spectroscopy (EDS, Oxford Instruments, AZtec).

#### 2.4.6. FTIR spectroscopy

The infrared spectra of MK, GO, and MK–GO hybrid were recorded using a Fourier transform infrared spectrometer (Perkin Elmer, Frontier FT-MIR) with a LiTaO_3_ (Lithium tantalite) detector and optical system with KBr beam splitter. All the spectra were obtained at a 2 cm^−1^ resolution over the wavenumber ranged from 4000 to 500 cm^−1^ by spectrometer through the employing KBr pellet method.

## 3. Results and discussions

### 3.1. Evaluation of the dispersion state of suspensions in water

Fig. S3† demonstrates the visual investigation of GO and MK–GO suspensions besides the result of their pH measurements in the deionized water. This image was taken about 1 h after the preparation of the samples. As can be seen, despite the acidic characteristic of GO dispersion, the MK–GO suspensions in water had relatively high pH values approximately in the range of 10.1–10.9, which may be mostly due to the partial dissolution of the chemical compounds present in MK (Table 1). Consequently, this can lead to the release of small amounts of alkali and earth alkali cations such as K^+^ and Ca^2+^.^38^ Also, it is obvious that the pH values of the suspensions will increase with increasing the MK/GO weight ratio which can be owing to the presence of the higher concentration of the aforementioned cations. Furthermore, while GO was quite stable and no traces of solid particles were recognizable in the suspension, all of the MK–GO samples were quickly settled down at the bottom of the suspensions so that their supernatant became transparent. Also, with the increase in the amount of MK, the MK–GO aggregates become denser and subsequently occupy less volume, as can be seen at the bottom of the suspensions. Accordingly, the observed behavior of MK–GO suspensions in water, compared with the pure GO, can be evidence for their interaction and should be investigated in deep.

**Fig. 1:**
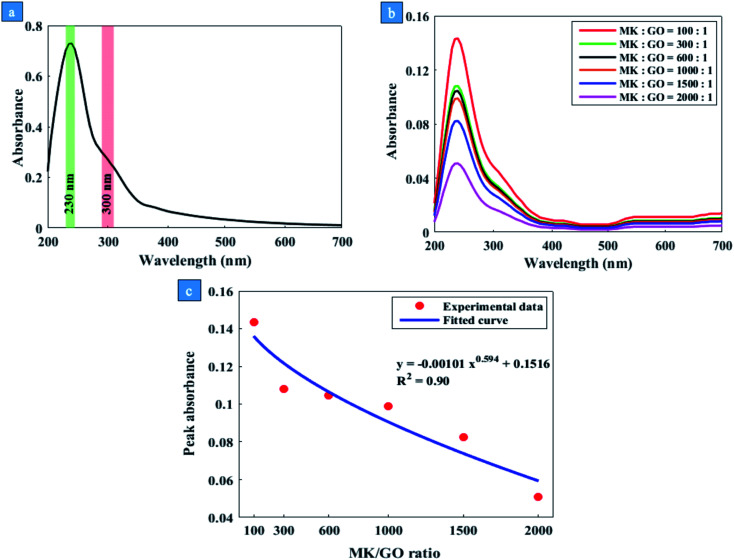
(a) UV-vis spectrum of GO in water, (b) the effect of different MK/GO weight ratios on the UV-vis spectra of GO and (c) fitting curve to the peak absorbance of MK–GO hybrid in water for different MK/GO weight ratios.

One of the most common methods to evaluate the dispersion degree of GO nanosheets in the host medium is measuring the UV-vis absorption. The mechanism of this method is based on the Beer–Lambert law^39,40^ that is displayed in eqn (1).1
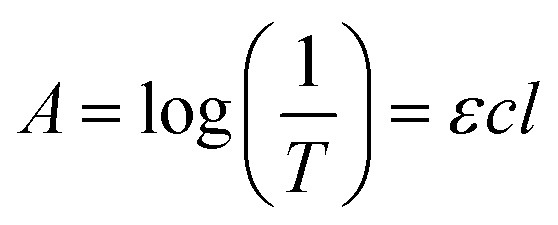
where *A* is the absorbance, *T* is the transmittance and equal to the ratio of the transmitted light beam intensity to the incident light beam intensity, *ε* is the molar absorption coefficient, *c* is the molar concentration of the dispersant, and *l* is the optical path length (1 cm). Based on the Beer–Lambert law, the intensity of the absorbed light is directly proportional to the concentration of the dispersant. It can be deduced from this law that the improvement in dispersion will lead to an increase in absorbance peak since only well-dispersed particles can absorb UV-vis light beam properly.^41^Fig. 1(a) depicts the UV-vis spectrum of GO dispersion in the deionized water. Two distinct characteristics have been specified in this spectrum, first located at the wavelength of 230 nm in the form of a peak that is due to the π–π* electron transitions of C=C bonds. Another one is a shoulder at the wavelength of 300 nm which is attributed to the n–π* electron transitions of C=O bonds. Fig. 1(b) represents the results of UV-vis spectroscopy of GO suspensions modified by MK for MK/GO (wt%) = 100, 300, 600, 1000, 1500, and 2000. As can be seen, the absorbance peaks of the samples containing GO and MK have been declined sharply compared with pure GO in water which was in accordance with the visual investigation results. These low adsorptions can be attributed to the very small amounts of residual GO nanosheets in suspensions that are not agglomerated by MK particles. This severe decrease in the peak absorbance of GO, as well as the agglomeration of MK–GO particles in aqueous solutions, confirmed the reaction among GO and MK particles in water.

**Fig. 2:**
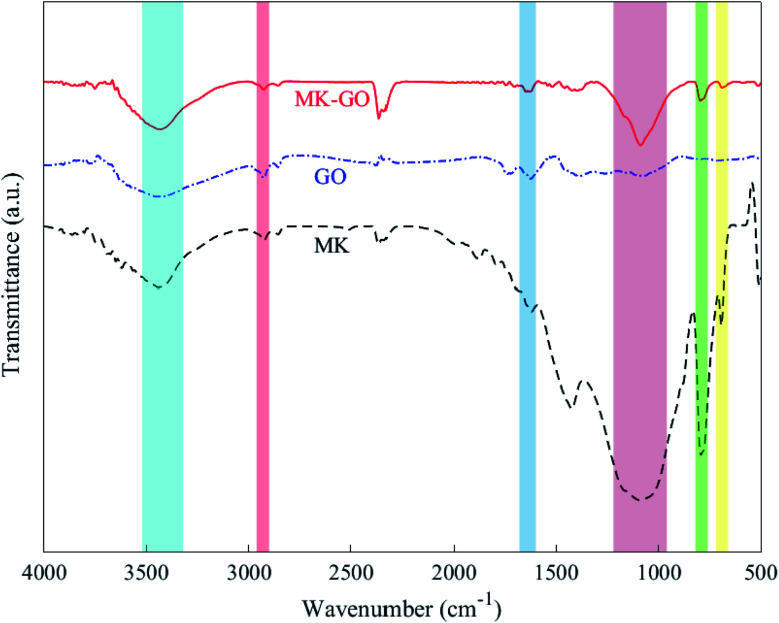
FTIR spectra of MK, GO, and MK–GO.

The probable reaction between GO and MK in water was investigated by employing Fourier transform infrared (FTIR) spectroscopy. Fig. 2 shows the FTIR spectra of MK, GO, and MK–GO. It is observed that the FTIR spectrum of the MK–GO sample has some peaks in common with the other spectra which have been featured by colorful regions. In the case of MK, the peaks observed at 694 and 792 cm^−1^ are attributed to the symmetric stretching vibration of Si–O–T (T = Al or Si) while the peak at 1090 cm^−1^ corresponds to the asymmetric stretching vibration of Si–O–T (T = Al or Si).^42–44^ Moreover, the –OH stretching vibration peak at 3440 cm^−1^ can be due to the defective dehydroxylation of kaolin during the calcination process.^43^ Meanwhile, the spectrum of MK–GO also exhibited two other peaks at 1648 and 2925 cm^−1^ which mainly can be attributed to the sp^2^ hybridized C=C bond and C–H bond in the structure of GO, respectively. Notably, the position of the C=C bond peak in MK–GO shifted to a larger wavenumber value compared with that one in GO, which is an indicator of the reaction between the graphene layer of GO and chemical components of MK as explained previously in the literature.^45,46^

Indeed, for interpretation of the UV-vis spectroscopy and also visual observation results of MK–GO suspensions in water, it seemed necessary to further investigate the chemical composition of MK. According to the XRF analysis of MK (Table 1), it can be seen that SiO_2_ and Al_2_O_3_ are the main chemical oxides constituting MK. Therefore, it can be concluded that the behavior of MK in different environments basically corresponds to the behavior of its SiO_2_ and Al_2_O_3_ compounds. Former research by Ren *et al.*^46^ revealed that the incorporation of SiO_2_ solid particles into the GO dispersion at the pH range of 10–11 had a negligible effect on the concentration of the residual GO in the supernatant. Afterward, this finding was confirmed by Lu *et al.*^23^ by investigating the UV-vis absorbance of GO and SF–GO suspensions in water. Based on their calculation, the peak absorbance of SF–GO suspension was just approximately 5 percent lower than that one in GO which could be due to the chemical composition of utilized SF in their study. However, Ren *et al.*^46^ reported that the incorporated Al_2_O_3_ solid particles significantly decreased the concentration of GO in the supernatant at a relatively high pH range of 10–11. In this alkaline environment, despite the strong electrostatic repulsion forces between the negatively charged particles of Al_2_O_3_ and GO, the deposition of GO on Al_2_O_3_ particles is assigned to the Al dissolution of Al_2_O_3_ and consequently the formation of Al(OH)_4_^−^ at the pH > 8.7.^46^ As Al(OH)_4_^−^ is a Lewis acid, the delocalized π electron systems of the graphene layer as a Lewis base can form electron donor–acceptor complexes with Al(OH)_4_^−^.^45^ Therefore, a strong surface complexation between GO nanosheets and Al(OH)_4_^−^ through the Lewis acid–base interaction contributed to the GO aggregation.^46^ Based on the aforementioned mechanism, it can be expected that the deposition of GO on the surface of MK increases with an increase in the value of MK/GO weight ratio due to a higher production of Al(OH)_4_^−^. Consequently, the concentration of GO in the supernatant of MK–GO suspension decreases significantly compared to pure GO suspension. As a result, the peak absorbance in the UV-vis spectra of the supernatants of MK–GO suspensions reduces in descending order from mix 100 to mix 2000 which is also obvious from the curve that is fitted to the results and shown in Fig. 1(c). As depicted in Fig. S4,† the well-dispersed GO in the supernatant of the centrifuged MK–GO suspension is featured by its slightly yellow color while the precipitated GO is specified by its dark brown color which accumulated on MK particles.

**Fig. 3:**
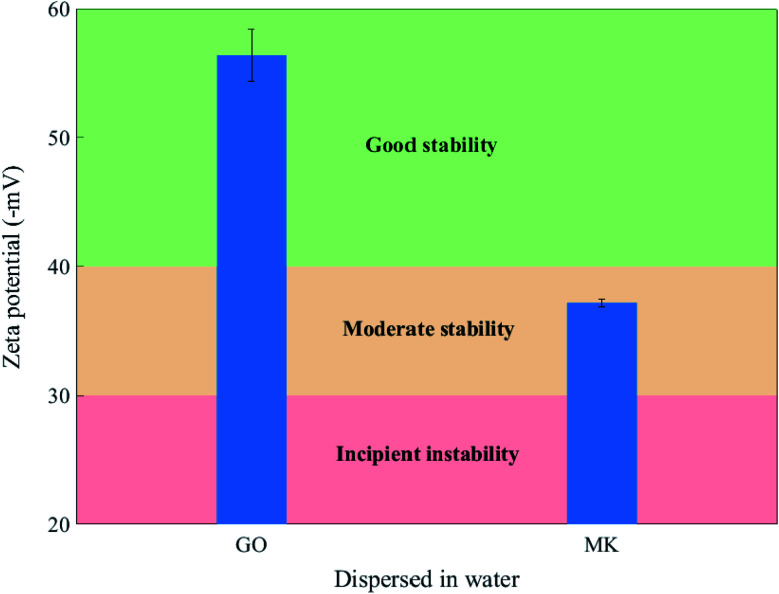
Zeta potential of GO and MK dispersed in water at the pH of 10.5.

Besides the previous experiments, zeta potential measurement was adopted in this work for evaluating the colloidal stability of MK, GO, and MK–GO suspensions based on the magnitude of their zeta potential values and also, more importantly, to investigate the adsorption process between MK and GO in water and SCPS. The zeta potential values of MK and GO suspensions in water at the pH of 10.5 are shown in Fig. 3. It can be seen that GO exhibited a high negative value of zeta potential (∼−56 mV) which is attributed to the deprotonation of the oxygen-containing functional groups grafted on the surface of GO such as hydroxyl (–OH) and carboxyl (–COOH).^47,48^ Consequently, the increased surface charge density of GO was the main reason for the good stability of GO because of generating strong electrostatic repulsion between GO nanosheets, which can dominate weak van der Waals attractive forces between them.^49^ On the other hand, MK had moderate stability in water with a negative zeta potential value of ∼−37 mV. This negative value stems from the progressive deprotonation of silanol (Si–OH) and aluminol (Al–OH) groups, which present on the surface of hydrolyzed MK particles to silanolate (Si–O^−^) and aluminolate (Al–O^−^) groups, respectively, at the relatively high pH value of 10.5.^50,51^ Hence, it can be inferred that MK particles possess anionic charge density on their surfaces and therefore can repel each other by electrostatic repulsive forces. Next, to further characterize, particle size analysis was performed on GO and MK suspensions in water, and the results are presented in Fig. S5.† As observed in Fig. S5(a),† the lateral size of GO nanosheets was in the range of 600–1400 nm with an approximate average size of 900 nm which was in good accordance with the reported values in the literature.^37^ Also, Fig. S5(b)† demonstrates that none of the dispersed MK particles had a diameter larger than 50 μm. Additionally, more than 80 percent of the dispersed MK particles possessed a diameter smaller than 10 μm, as well, the average size of MK particles was approximately 5.5 μm.

**Fig. 4:**
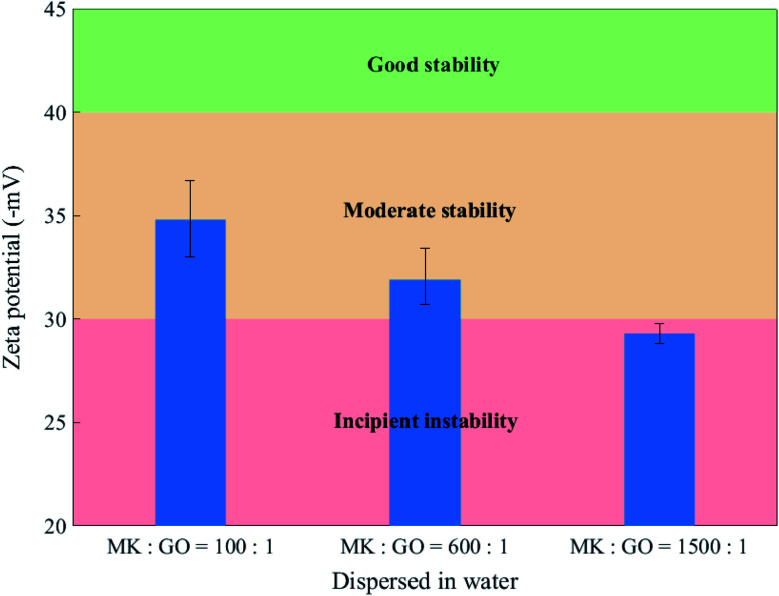
Zeta potential of MK–GO suspensions with different MK/GO weight ratios dispersed in water at the pH of 10.5.

The zeta potential values of the MK–GO hybrid suspensions for MK/GO weight ratios of 100, 600, and 1500 are presented in Fig. 4. In this case, the variation of the zeta potential values in comparison with the individual GO and MK suspensions illustrates that GO has a tendency to be adsorbed on the surface of MK particles despite the electrostatic repulsive forces between them. Similar to the observations reported and explained by de Reese *et al.*^52^ and Plank *et al.*,^53^ it seems necessary to interpret the adsorption of GO on MK particles from thermodynamics viewpoint. Practically, the adsorption of GO on the surface of MK particles is a spontaneous process if the Gibbs free energy of adsorption is negative in sign. Based on the Gibbs–Helmholtz equation (eqn (2)), it can be concluded that the Gibbs free energy (Δ*G*) depends on entropic (Δ*S*) and/or enthalpic (Δ*H*) contributions due to the adsorption process. Also, it is obvious that the Gibbs free energy is negative when the enthalpic and/or entropic terms decreases and/or increases, respectively, as a result of the adsorption process.

2 Δ*G* = Δ*H* − *T*Δ*S*

**Fig. 5:**
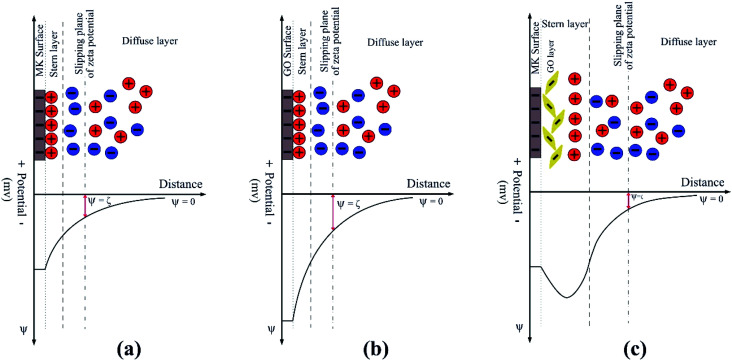
Schematic illustration of the electrochemical double layer of (a) MK, (b) GO and (c) MK–GO dispersed in water at the pH of 10.5.

Generally, in the case of GO adsorption on similarly charged surfaces of MK particles, three main energy contributions can participate which are: (1) the electrostatic repulsion between GO and MK and also between GO nanosheets themselves which are adsorbed on the MK particles; (2) the H-bonding between MK particles and oxygen-containing functional groups of GO in MK–GO hybrid as can be inferred from the position shift of –OH stretching vibration peak to a lower wavenumber value compared to those of GO and MK (Fig. 2); and (3) the increase in entropy due to the release of a large amount of ions and water molecules into the suspension by MK and GO when the adsorption process occurs.^9^ The first and the second term corresponds to the enthalpic contribution (Δ*H*) while the third one belongs to the entropic contribution (Δ*S*). Moreover, it should be pointed out that the first term has an inhibiter role during the adsorption process, whereas the second and the third ones are beneficial to progress the adsorption. Therefore, the adsorption of GO on the surface of MK particles occurs only if the favorable contributions overcome the contrary ones and stops when the electrostatic repulsive forces dominate the released energy from the hydrogen bonding and the increase in entropy. Based on the aforementioned mechanisms, it can be deduced that the adsorption of GO on the surface of MK particles is mainly propelled by a huge gain in entropy which has the dominant effect compared with the electrostatic repulsive forces. Hence, it can be expected that the adsorption of GO on MK particles can be promoted by an increase in the MK/GO weight ratio due to the higher release of ions into the suspension; while the electrostatic repulsive forces decrease continuously based on the zeta potential values of MK–GO hybrid. It is noteworthy mentioning that although the adsorption of GO on MK particles makes the MK stern layer more negatively charged in comparison with the one in the individual MK, however, it should be pointed out that the absorbed GO moves the shear plane of zeta potential to a farther distance away from the MK surface where the zeta potential has a less negative value.^54,55^Fig. 5 depicts the simplified double layer of MK, GO, and MK–GO when dispersed in water. Additionally, the outcomes of the particle size analysis of MK–GO suspensions are shown in Fig. S6.† The results revealed that the average particle size of MK–GO suspensions was numerically equal to 8.1 μm, 8.5 μm, and 14.4 μm for MK/GO weigh ratios of 100, 600, and 1500, respectively. Accordingly, the shift in particle size distribution toward the upper values of diameter could be evidence for the higher adsorption of GO on MK particles with an increase in the dosage of MK, which leads to the following alterations: (1) more surface complexation between GO and MK due to the higher production of Al(OH)_4_^−^; (2) increase in entropy by releasing larger amounts of ions into the suspension; (3) decrease in enthalpy through lowering the electrostatic repulsive forces; (4) providing more surface area of MK, which leads to an increase in the exposed area of MK for GO adsorption as a result of the approximate equivalency between the surface area of the incorporated MK and GO as explained by Li *et al.*^20^

**Fig. 6:**
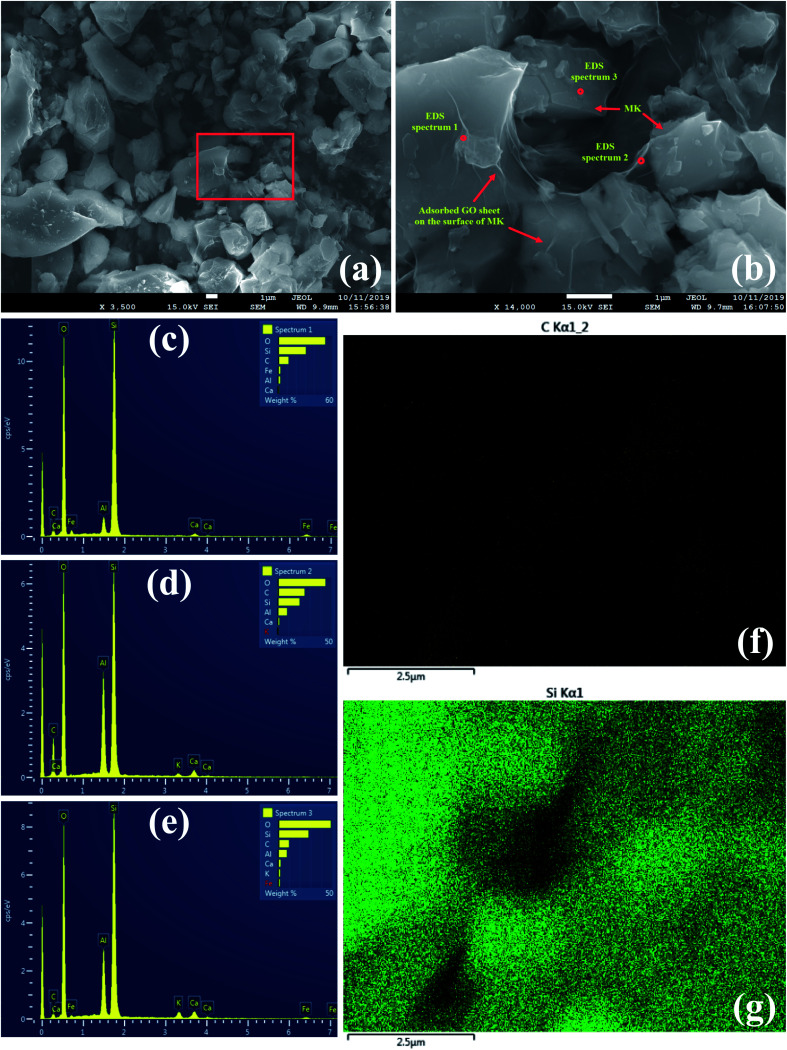
SEM images of MK–GO suspension in water with (a) 3.5k× and (b) 14k× magnification alongside its EDS (c) spectrum 1, (d) spectrum 2 and (e) spectrum 3, corresponding elemental distribution maps of (f) carbon and (g) silicon of MK–GO suspension.

Along with the previous experiments, SEM imaging was also conducted on the MK–GO suspension in water to further investigate the interaction between MK and GO in an infinitesimally small scope. SEM micrographs alongside the EDS analysis results are presented in Fig. 6. It is observed that unlike the study done by Lu *et al.*,^23^ in this case, GO sheets adsorbed on the surface of MK particles as a thin layer linking them with each other which can be responsible for the increased size of MK–GO suspensions relative to the individual MK suspension based on the particle size analysis results. Moreover, the elemental composition of MK–GO suspension in three different points was determined using the EDS analysis. The presence of MK and GO in the mentioned points were detected because of the existence of silicon (Si), aluminum (Al), oxygen (O), and carbon (C) dominant peaks. Furthermore, the low-intensity peaks of Ca, K, and Fe could be attributed to the partial dissolution of the chemical compounds of MK (Table 1) in water. Besides, the EDS elemental mapping of carbon and silicon elements that correspond to the GO nanosheets and MK particles, respectively, in their hybrid in water are also depicted.

### 3.2. Evaluation of the dispersion state of suspensions in SCPS

Guarantying the good dispersion of GO nanosheets in high alkaline environment of a freshly mixed cement composite is an essential prerequisite for fabricating the GO modified cement-based composites with better properties. Indeed, although investigating the dispersion behavior of MK–GO suspensions in water is necessary to illuminate their interaction in the mix water before blending with cement, the evaluation of the dispersion status of MK–GO suspensions in SCPS has greater importance to determine whether MK particles are capable of ensuring the good dispersion of GO in alkaline cementitious environments or not. Hence, to simulate the interaction between the MK–GO suspension and alkaline cementitious environments, dispersion characterizations similar to those in water were conducted on MK–GO suspension in SCPS. The UV-vis absorbance of GO and also MK–GO suspensions for MK/GO (wt%) of 100, 300, 600, 1000, 1500, and 2000 in SCPS were very low and close to zero due to the high level of agglomeration of GO nanosheets. This severe agglomeration is obviously observable from the visual inspection image demonstrated in Fig. S7.† It should be pointed out that this image was taken a few minutes after the contact between prepared suspensions and SCPS. The result of pH measurements demonstrates that all the suspensions in SCPS exhibit high alkalinity in the range of 13.10–13.25 which is due to the existence of significant amounts of monovalent and divalent cations and anions such as Na^+^, K^+^, Ca^2+^, and OH^−^ in the alkaline environment of SCPS.^16,56^ Based on the pH values in SCPS, it can be concluded that the increase in the MK/GO weight ratio has a minor impact on reducing the pH values of MK–GO suspensions in SCPS at an early age. Hence, it reveals that MK has negligible potential for quite consuming saturated Ca^2+^ cations and also abundant OH^−^ anions as main obstacles against desirable dispersion of GO in SCPS through its pozzolanic activity. Furthermore, the transparent supernatant of MK–GO suspensions in SCPS and consequently their UV-vis absorption results can be mainly due to the combined effects of the Al_2_O_3_ compound which exists in MK and also the presence of Ca^2+^ cations in SCPS. According to a previous study,^57^ the threshold concentration of the Al-systems for GO aggregation was approximately 0.01 mmol L^−1^. This value is much less than (∼twentieth) that one exists in the alkaline solution owing to the dissolution of the Al_2_O_3_ compound of MK over a reaction time of 2 h since the fabrication of suspensions.^46^ On the other hand, Ca^2+^ cations because of their higher valence, compared to Na^+^ and K^+^, have a stronger binding capacity with the oxygen-containing functional groups of GO which makes more neutralization of the GO negative surface charge.^46^ Therefore, based on the abovementioned information, it can be expected that the rate of GO aggregation and subsequent sedimentation in MK–GO suspensions must be higher than that one in GO due to the existence of critical concentration of Al, as can be seen from their visual investigation image. Indeed, MK cannot play the role of an obstacle against GO agglomeration in SCPS and, oppositely, can act as an agglomerative agent. Therefore, based on the pH measurements and visual investigation image, the hypothesis that MK as a pozzolanic material can lower down the Ca^2+^ concentration through the pozzolanic reaction and rectify the dispersion of GO nanosheets in SCPS cannot be authentic. Similar results have been reported by Lu *et al.*^58^ about silica fume by performing atomic absorption spectroscopy (AAS) measurements. They stated that silica fume cannot cause a remarkable change in the concentration of calcium divalent cations in the cement pore solution.

**Fig. 7:**
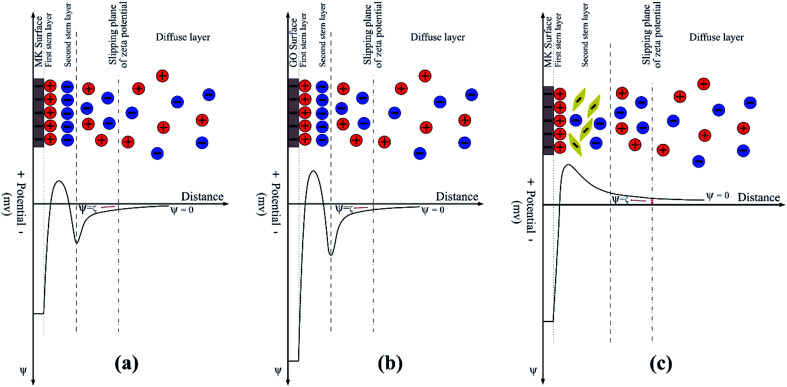
Schematic illustration of the electrochemical double layer of (a) MK, (b) GO and (c) MK–GO dispersed in SCPS at the pH of 13.2.

The results of zeta potential measurements of the suspensions in SCPS are listed in Table 4. As can be seen, the zeta potential value of MK suspension in SCPS is decreased significantly to a less negative value (∼−2.8 mV) compared with the one in water (∼−37 mV) and approximately approached zero. It should be pointed out that the surface charge and subsequently the zeta potential of MK suspension in SCPS result from several simultaneous interactions between the surface of MK particles and the different ions present in this environment. Due to the high alkalinity of SCPS, most of the silanol (Si–OH) and aluminol (Al–OH) groups on the surface of MK particles deprotonate to silanolate (Si–O^−^) and aluminolate (Al–O^−^) groups, respectively. Hence, it can be concluded that the anionic charge density on the surface of MK particles increases noticeably, which can act as a potential anchoring site for the cations that exist in SCPS through the electrostatic attraction forces.^50^ The major cations in SCPS are Na^+^, K^+^, and Ca^2+^ which are abundant in it. Because of their multivalent property, Ca^2+^ cations can be adsorbed on the high negatively charged surface of MK particles due to the stronger electrostatic attraction between them and therefore are more effective than Na^+^ and K^+^ in neutralizing the zeta potential value of MK. This way, a layer of Ca^2+^ cations is formed on the surface of MK particles which is the first stern layer, as reported by a former study.^59^ Indeed, the formation of this layer renders the surface of MK positively charged through the charge neutralization/reversal mechanism. Consequently, this positive layer can adsorb sulfate anions that exist in SCPS, thus forming a second stern layer of anions that alters the surface charge of MK to negative. However, the formation of this second stern layer and also suppression of the electrochemical double layer of MK by Na^+^ and K^+^ cations^57^ result in a low negative value of zeta potential close to zero for MK particles. Fig. 7(a) represents the schematic illustration of the electrochemical double layer existing on the surface of MK particles when dispersed in SCPS. In this case, it could be expected that the van der Waals attractive forces between MK particles dominate extremely low repulsive forces. Therefore, MK particles aggregate by adsorbing each other which causes the average size of MK particles in SCPS (∼18 μm) significantly be larger than those in water (∼5.5 μm) as shown in Fig. S8(a).†

**Table 4:** Zeta potential of the samples in SCPS

Sample name	MK : GO weight ratio	Zeta potential (mV)
MK	—	−2.8 ± 0.7
GO	—	−0.6 ± 0.1
MK–GO	100 : 1	1.8 ± 0.3
MK–GO	600 : 1	3.1 ± 0.4
MK–GO	1500 : 1	7.3 ± 1.5

Similar to MK, the obtained result for the zeta potential of GO in SCPS evidenced its drastic colloidal instability in this highly alkaline environment. Actually, this instability is mainly due to the significant reduction in electrostatic repulsive forces between GO nanosheets immediately after their contact with SCPS. Based on the previous studies,^57,60^ several mechanisms for explaining the considerable decrease in zeta potential values of GO nanosheets have been developed which are as follows: first, the divalent Ca^2+^ cations are adsorbed on the high negatively charged surface of GO due to the strong electrostatic attraction between them. Second, the adsorbed Ca^2+^ cations neutralize the negative charges of GO because of their strong binding capacity with oxygen-containing functional groups of GO. Third, the monovalent cations such as Na^+^ and K^+^ can compress the electrochemical double layer of GO and consequently decrease its zeta potential value. It is worth mentioning that in this case, a double stern layer similar to that one in MK is formed; its schematic illustration is shown in Fig. 7(b). Furthermore, the particle size analysis of GO in SCPS also elucidated its aggregation in this environment. As shown in Fig. S8(b),† the average particle size of GO in SCPS (∼110 μm) significantly increased compared to that in water (∼0.9 μm) which is also in agreement with previous studies.^37^

Compared with the individual MK and GO suspensions in SCPS, it can be seen that the zeta potential values of MK–GO suspensions in SCPS are positive in sign and slightly increase with an increase in the MK/GO weight ratio. Here, the high negatively charged surface of MK particles provides abundant potential sites for adsorbing cations which are present in SCPS, especially Ca^2+^, *via* electrostatic attraction forces. Therefore, a layer of multivalent cations is formed on the surface of MK which renders its surface positively charged. It is noteworthy mentioning that by increasing the amount of MK, more negative anchoring sites can be made and subsequently more polyvalent cations can be adsorbed which means a higher value of the positive charge is attained on the surface of MK particles. Then, through the mediation of this positive stern layer, MK particles adsorb GO nanosheets and sulfate anions which leads to the formation of the second stern layer. It should be indicated that Ca^2+^ cations on the surface of MK particles interact with GO by coordination and consequently complexation with its deprotonated oxygen-containing functional groups^61^ and also *via* cation–π binding with its basal plane.^57^ In this case, the sign change of zeta potential values is due to the formation of more complexations, which is the result of an increase in the cationic charge density of the first stern layer. The schematic representation of this situation is depicted in Fig. 7(c). In summary, in this case, it can be expected that the mediation effect of Ca^2+^ cations interlocks the MK particles and GO nanosheets and consequently leads to the formation of their cross-links in SCPS.^62^ As mentioned above, an increase in the amount of MK makes the cationic charge density of the first stern layer increases significantly, thus attracting much more GO nanosheets and sulfate anions. As a result, it can be concluded that the average size of MK–GO aggregates increases because of higher cross-linking of GO nanosheets and MK particles by Ca^2+^ cations which is obvious in the particle size analysis results of the various MK–GO suspensions presented in Fig. S9.† Nevertheless, it is noteworthy mentioning that the lateral dimension of MK modified GO aggregates with the average size in the range of 12–15 μm exhibited a significant reduction in comparison to individual GO aggregates with the average size of 110 μm.

**Fig. 8:**
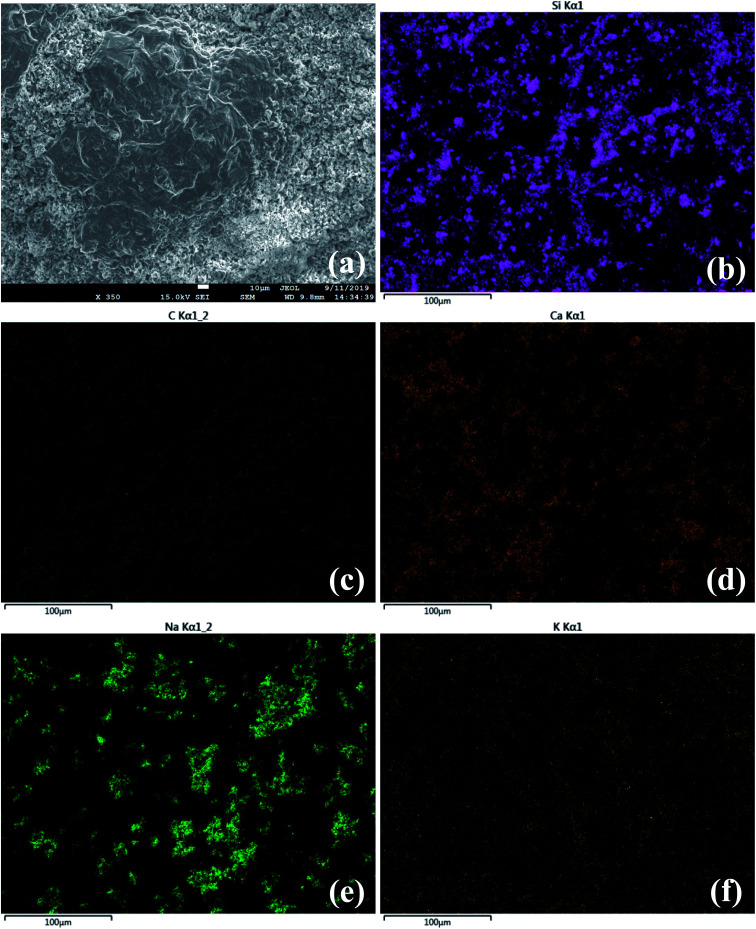
SEM image of MK–GO suspension in SCPS at low magnification (a) alongside its corresponding elemental distribution maps of (b) silicon, (c) carbon, (d) calcium, (e) sodium and (f) potassium.

Finally, besides the previous experiments in SCPS, for further investigation of the dispersion state of MK–GO sample, SEM imaging was employed. SEM micrographs of MK–GO suspension in SCPS are shown in Fig. 8. As can be seen, EDS mapping analysis of Si and C elements clearly demonstrated the aggregation of MK–GO hybrid in SCPS, because of the non-uniform distribution of the abovementioned elements with many blank dark spaces that are obvious in their micrographs. On the other hand, based on the results of EDS elemental mapping, compared to the distribution pattern of K^+^ and Na^+^ cations, the pattern of Ca^2+^ cations was more similar to those of Si and C elements. This observation could be evidence for the crucial role of Ca^2+^ cations in the agglomeration of MK–GO suspension in SCPS because of their cross-linking effects as reported by previous studies.^19,62^

Eventually, based on the obtained results for MK–GO suspension in SCPS, it is deduced that the MK was not able to effectively restrict the GO agglomeration in SCPS, and even it may accelerate the agglomeration and sedimentation of GO. Therefore, it can be expected that immediately after the mixing of MK–GO suspension with cement grains to make cement composite, due to the hydration of cement particles in the alkaline environment of the freshly mixed cement composite, the formed MK–GO agglomerates may cause the formation of weak zones in the cement composite before its hardening. This way, the enhancing effects of MK–GO on mechanical and transport properties of cement composites can be restricted significantly owing to their non-uniform dispersion in the cement matrix. However, the improvement in the dispersion status of MK–GO hybrid in cement composites using various methods such as the incorporation of surfactants can be a potential remedy for making more durable cement composites with enhanced mechanical properties. Actually, among the different kinds of surfactants which have been experimentally and computationally examined in the previous studies,^9,18^ the polycarboxylate ether (PCE) based generation exhibits the best capability to prevent GO agglomeration in cement composites. Nevertheless, the efficiency of PCE to improve the dispersion of pozzolan-modified GO suspensions in fresh cement matrix has not been deeply investigated yet and should be further evaluated in future works.

## 4. Conclusions

In this paper, an experimental investigation on the dispersion of GO in water and simulated cement pore solution with the incorporation of MK was conducted using different characterizations. Based on the UV-vis absorption results, the increase in the amount of MK had an inverse effect on the concentration of well-dispersed GO nanosheets in water which could be attributed to the interaction between the Al_2_O_3_ compounds of MK and GO nanosheets. The evidence of this interaction was observed in the FTIR spectrum of MK–GO suspension in water. Also, the colloidal stability of MK–GO samples was worse than the individual MK and GO ones in water, based on their less negative values of zeta potential and also visual investigation images. Based on the Gibbs–Helmholtz thermodynamic definition, the adsorption of the stable GO nanosheets on the surface of MK particles could be responsible for the obtained results of the zeta potential measurements. Besides, the particle size analysis revealed that the adsorption amount of GO was directly proportional to the dosage of MK. On the other hand, in the case of SCPS, almost all of the conducted experiments on the MK–GO hybrid showed that different dosages of MK particles were incapable of preventing GO agglomeration which elucidated that MK cannot restrict the disadvantageous impacts of Ca^2+^ cations on GO dispersion. Therefore, despite the mechanisms discussed in previous studies, MK cannot play as a dispersive agent for GO in SCPS and consequently in the fresh cement matrix. The result of this study revealed that there can be some complex interactions between MK and GO in different mediums which should be investigated in deep prior to their incorporation in cement composites.

## Conflicts of interest

There are no conflicts to declare.

## Supplementary Material

RA-011-D1RA01504D-s001
