# Supplementary material for: Evaluation of the dispersion of metakaolin–graphene oxide hybrid in water and cement pore solution: can metakaolin really improve the dispersion of graphene oxide in the calcium-rich environment of hydrating cement matrix?
Source: RSC Adv. 2021 May 24;11(30):18623–36. doi: 10.1039/d1ra01504d (PMC9033477; doi:10.1039/d1ra01504d)
Supplement: RA-011-D1RA01504D-s001 [file RA-011-D1RA01504D-s001.pdf]

Electronic Supplementary Material (ESI) for RSC Advances.
This journal is © The Royal Society of Chemistry 2021

Electronic supplementary information (ESI)

Evaluation of the dispersion of metakaolin-graphene oxide hybrid in water and cement pore solution: Can metakaolin really improve the dispersion of graphene oxide in the calcium-rich environment of hydrating cement matrix?

Kasra Amini [1], Siavash Soleimani Amiri [1], Ali Ghasemi [1], Sajjad Mirvalad [2]*, Asghar Habibnejad Korayem [3]*

[1] MSc student, School of Civil Engineering, Iran University of Science and Technology, Tehran, Iran

[2] Assistant Professor, School of Civil Engineering, Iran University of Science and Technology, Tehran, Iran

[3] Associate Professor, School of Civil Engineering, Iran University of Science and Technology, Tehran, Iran

** Corresponding authors: Email: mirvalad@iust.ac.ir, and ahkorayem@iust.ac.ir*

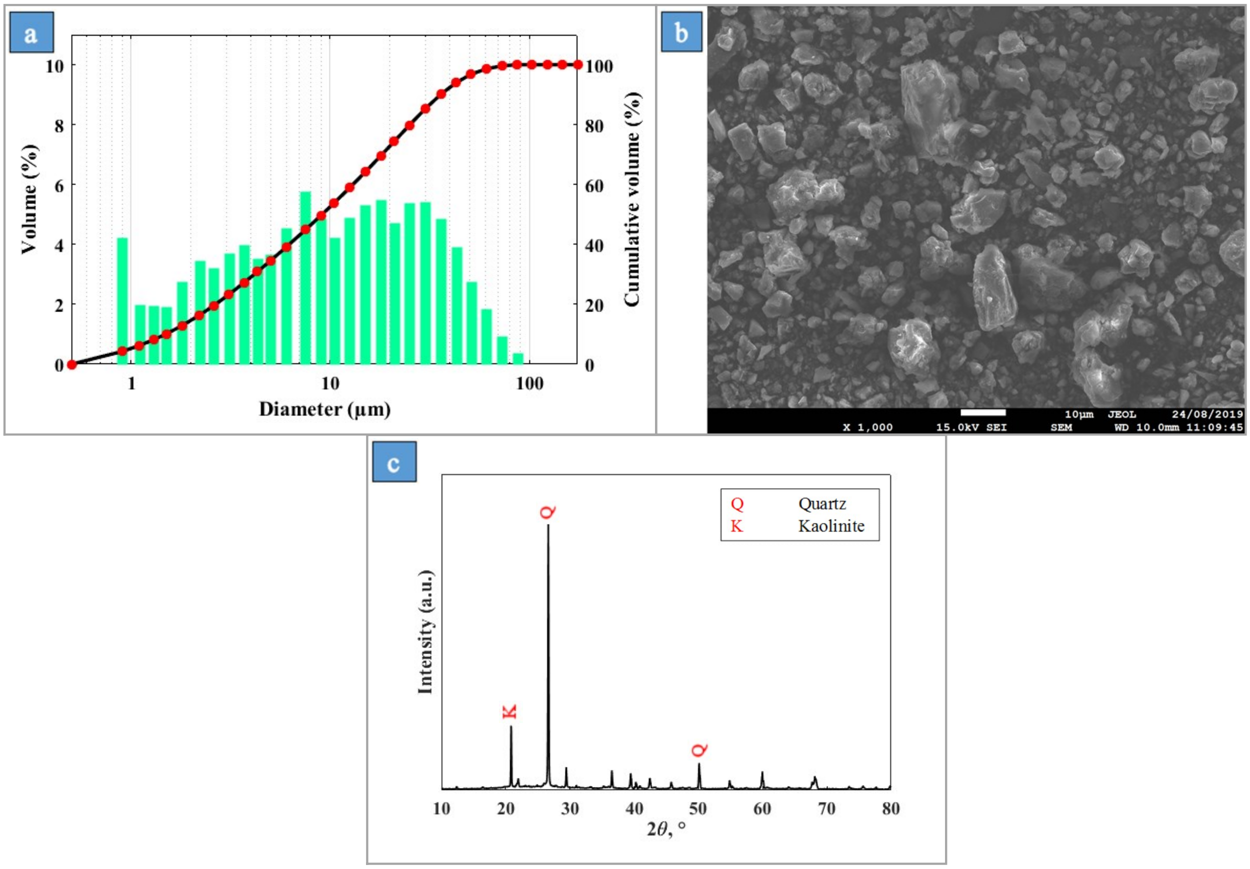

Fig. S1. (a) Particle size distribution (b) SEM image and (c) XRD pattern of metakaolin solid particles.

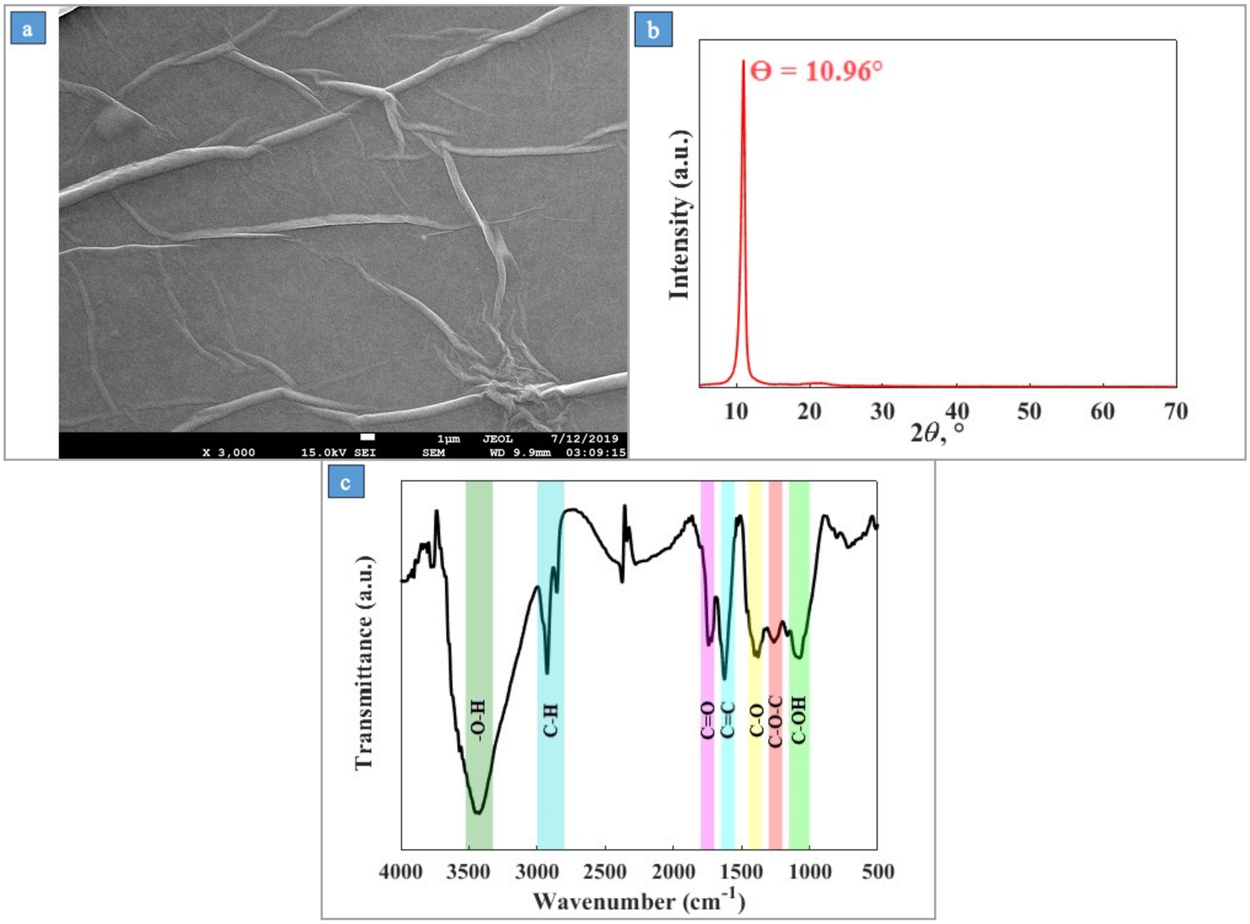

Fig. S2. Characterization of as-received GO (a) SEM micrograph (b) XRD pattern and (c) FTIR spectrum.

Table S1

The mix design of the prepared SCPS.

Used chemical (g/l)			
$CaSO_4.2H_2O$	$Ca(OH)_2$	KOH	NaOH
27.55	Saturated	22.44	8.00

Table S2

The result of ICP-OES analysis of SCPS.

Elemental concentration (ppm)				
K	Na	Ca	Si	Fe
7518.8	3943.4	780.05	1.6	0.01

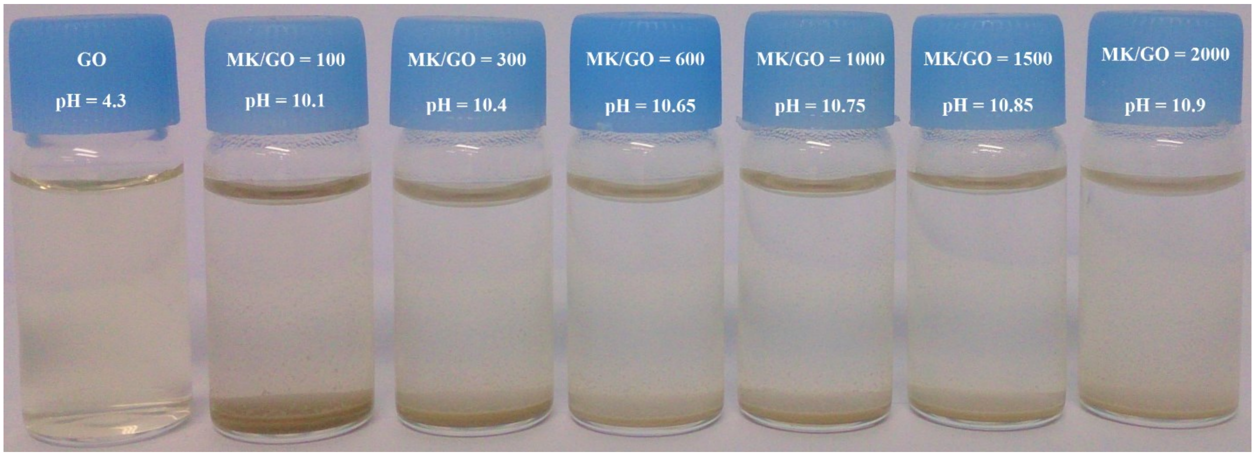

Fig. S3. Visual investigation of GO and MK-GO suspensions in water.

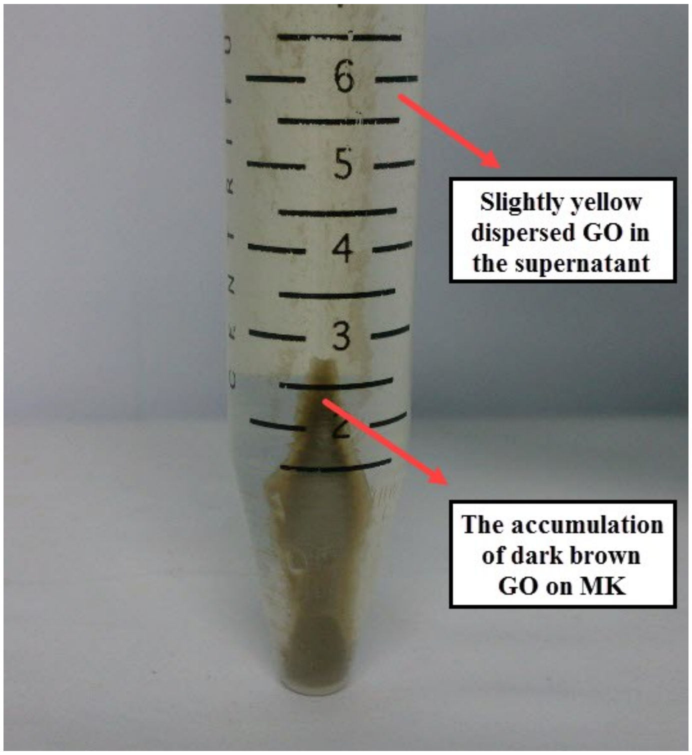

Fig. S4. Two-phase system of MK-GO suspension due to the centrifuging treatment, prior to the UV-vis characterization.

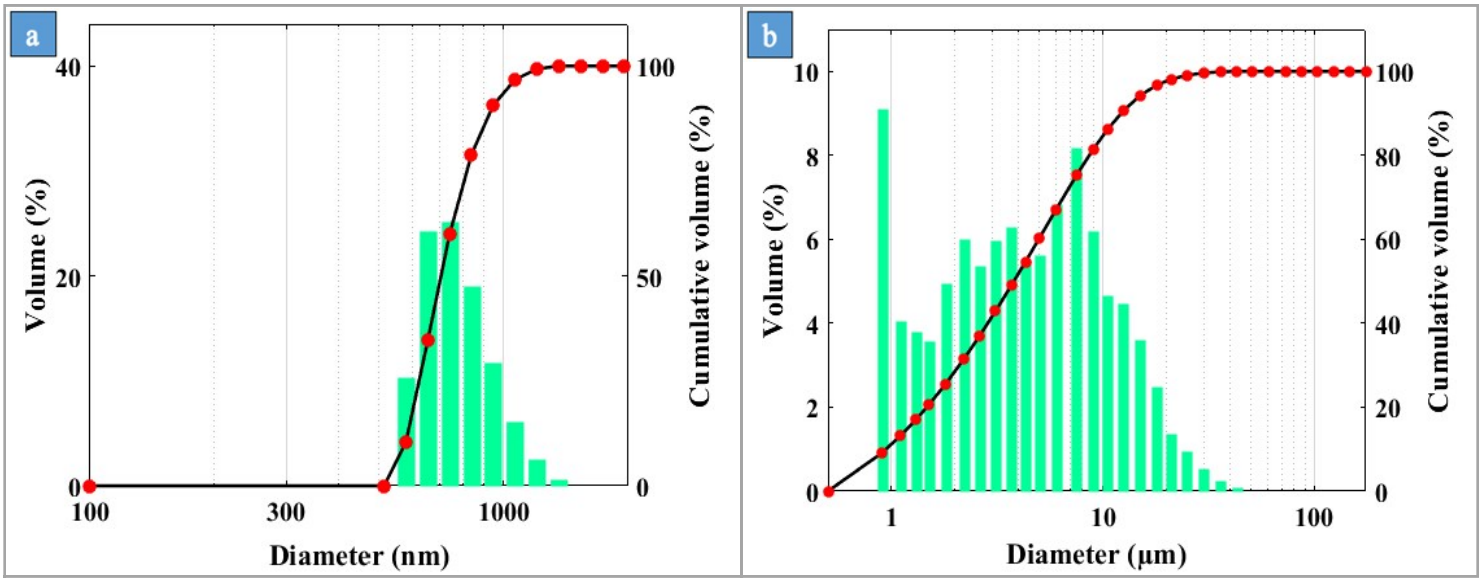

Fig. S5. Particle size distribution of (a) GO suspension and (b) MK suspension in water.

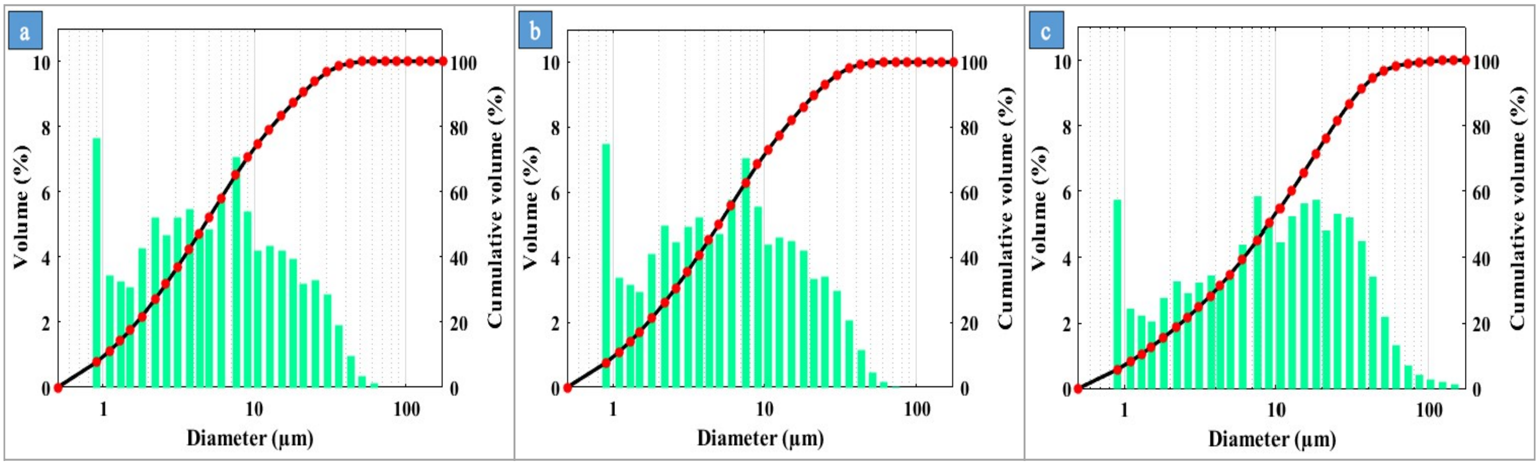

Fig. S6. Particle size distribution of MK-GO suspensions with different MK/GO weight ratios (a) MK/GO = 100 (b) MK/GO = 600 and (c) MK/GO = 1500 in water.

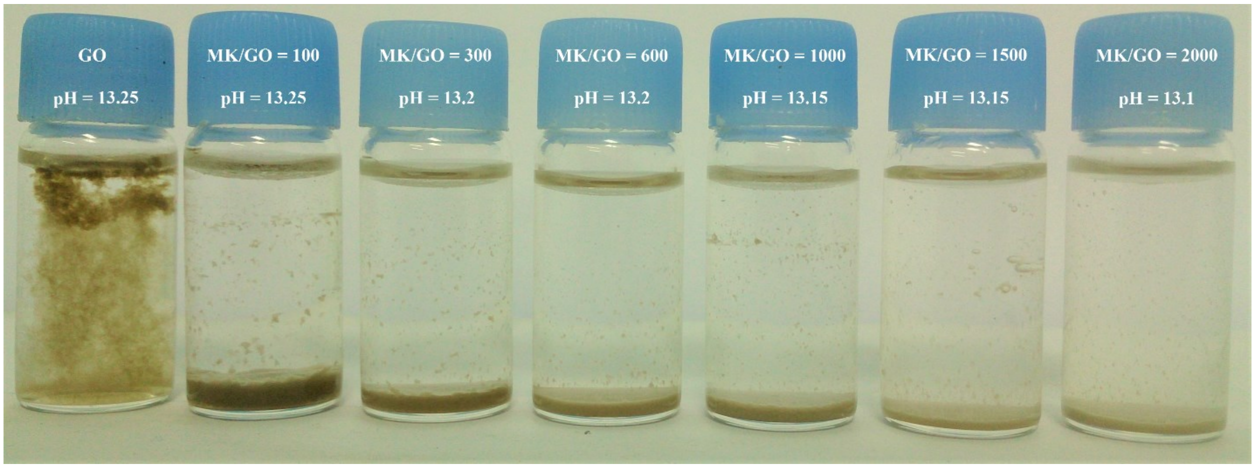

Fig. S7. Visual investigation of GO and MK-GO suspensions in SCPS.

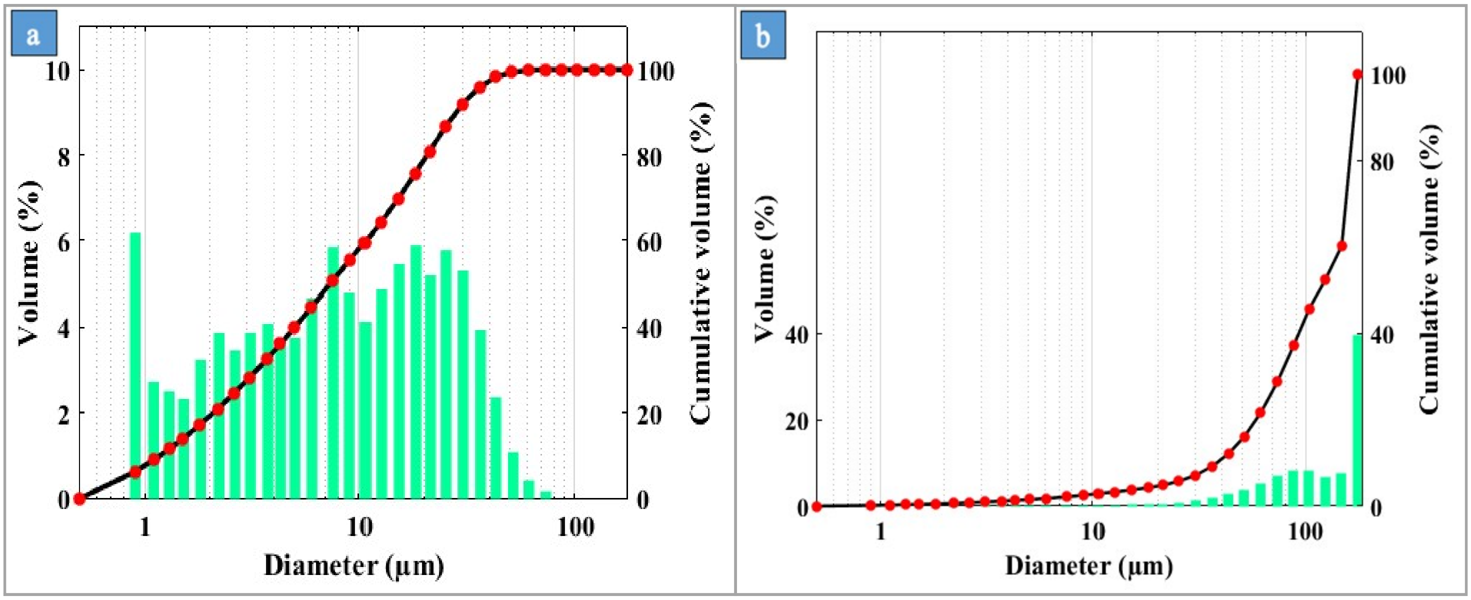

Fig. S8. Particle size distribution of (a) MK suspension and (b) GO suspension in SCPS.

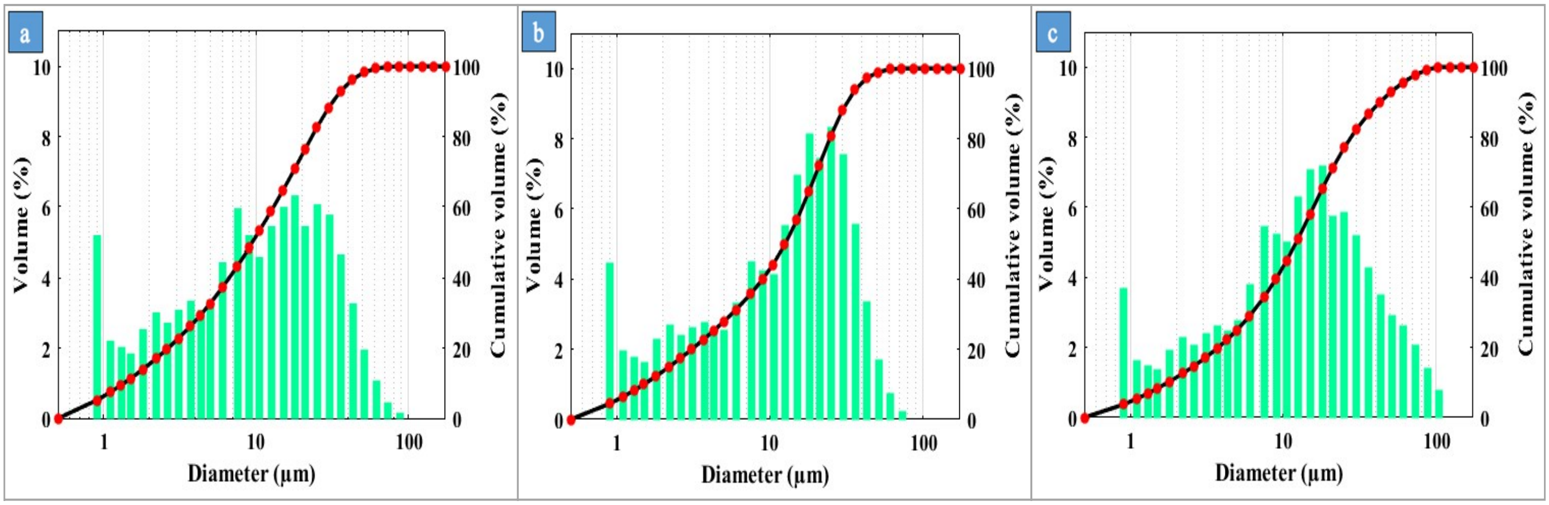

Fig. S9. Particle size distribution of MK-GO suspensions with different MK/GO weight ratios (a) MK/GO = 100 (b) MK/GO = 600 and (c) MK/GO = 1500 in SCPS.
